# Optimizing teduglutide treatment regimens in children with short bowel syndrome

**DOI:** 10.3389/fped.2026.1898958

**Published:** 2026-07-29

**Authors:** Qing Zhang, Min Hou, Meihua Jin, Xue Yang, Yang Li, Xiaoting Bi, Fang Liu, Yao Liu

**Affiliations:** 1 Department of Pharmacy, Daping Hospital, Army Medical University, Chongqing, China; 2 Department of Pharmacy, Chongqing Traditional Chinese Medicine Hospital, Chongqing, China

**Keywords:** children, optimize, PN independence, short bowel syndrome, teduglutide

## Abstract

Short bowel syndrome (SBS) is the main cause of intestinal failure (IF) in children, leading to severe malabsorption and dependence on parenteral nutrition (PN). The glucagon-like peptide-2(GLP-2) analogue teduglutide (TED) has emerged as the disease-specific treatment strategy capable of stimulating intestinal adaptation to promote PN reduction. In recent years, new randomized pediatric trials and growing real-world experience show that TED enables complete enteral autonomy in up to 30% of patients. This rate is much higher than the 16% reported in a 2022 systematic review of 14 pediatric studies, indicating that new research have generated different findings that can help optimize TED treatment regimens. These findings suggest that children with SBS who have significant baseline differences in PN dependence (lower PN volume, lower PN dependency index [PNDI], and fewer infusions per week) can benefit the most from teduglutide treatment. Determining the optimal timing to initiate TED for children with SBS requires individualized strategies rather than a universal recommendation. In addition, long-term administration of TED(at least one year before reassessing treatment response) is required in the treatment of SBS-IF to achieve the best therapeutic effect, which is essential for optimizing TED treatment regimens in children.

## 1 Introduction

SBS is a complex disease that occurs due to the physical loss or the loss of function of a portion of the small and/or large intestine (1), characterized by diarrhea, high ostomy output, malnutrition, and dehydration (2). As a result, patients with SBS have a decreased ability to absorb nutrients (3). In children, the main causes of SBS are congenital and perinatal diseases, such as necrotizing enterocolitis (NEC), malrotation leading to midgut volvulus, abdominal wall defects (gastroschisis) and intestinal atresia (4). The worldwide incidence of children with SBS is difficult to ascertain due to the lack of a comprehensive database.

SBS is the leading cause of intestinal failure (IF) in children and the potential cause of varying degree of nutrition support (5, 6). Most patients with SBS-IF cannot absorb sufficient nutrients through the gastrointestinal route and require intravenous supplementation of nutrients to maintain their nutritional status, making parenteral nutrition(PN) a critical treatment modality for this population (7). The use of PN reduces mortality and improves the overall outcomes. However, long-term PN use is linked to the development of multiple complications, including liver disease, catheter-associated malfunction and bloodstream infections (5). Therefore, the core therapeutic goal for children with SBS-IF is to maximize residual intestinal absorptive capacity, thereby reducing or eliminating reliance on parenteral support.

In recent years, glucagon-like peptide 2 (GLP-2) and its analogs have attracted interest from researchers as a potential therapeutic option for SBS-IF(2). Its clinical efficacy for SBS-IF has been confirmed in multiple clinical and real-world studies (8). TED is the first GLP-2 analog developed and marketed for SBS-IF, with a half-life of 2–3 h that requires daily administration. TED activates GLP-2 receptors in the gut and stimulates the secretion of insulin-like growth factor, nitric oxide and keratinocyte growth factor, thereby promoting epithelial cell proliferation, inhibiting apoptosis, accelerates injury healing, increases intestinal blood flow, decreases gastrointestinal motility (9), and reduces intestinal mucosal inflammation (10, 11). TED can restore the structural and functional integrity of the intestinal tract by promoting mucosal growth and slowing gastric emptying and secretion, thereby increasing the height of villi and the depth of crypts in the small intestinal mucosa, promoting nutrient absorption and enteral independence from parenteral nutrition. These effects collectively enhance the absorption of fluids and nutrients in patients with SBS-IF (12). Teduglutide is now FDA approved for the management of patients one year of age and older with SBS who are dependent on PN, or intravenous fluid.

A 2025 systematic review and meta-analysis investigating the effects of teduglutide for children with SBS only evaluated the impact of teduglutide on infection and gastrointestinal adverse events in children with SBS (13). A 2022 systematic review that included 14 studies of patients under 18 years of age with SBS-IF revealed that 16.14% of patients treated with teduglutide achieved enteral autonomy after a median of 24 weeks of treatment (IQR: 24–48 weeks) (14). However, this proportion is much lower than that reported in most studies published after 2022 (15–19)(29%–36%). Additionally, the initiation age of TED in the 2022 review was much older than that reported in recent studies (20)(4–12 months) and TED treatment duration was much shorter than that reported in recent studies (16–19, 21)(48–120.8 weeks), indicating that newer research have produced different results. This manuscript presents a narrative review of newly available evidence on the TED use for SBS in children. It focuses on the associations between patient baseline characteristics and achievement of enteral autonomy, as well as the optimal initiation time and duration of TED, to maximize benefits for children with SBS-IF who are capable of achieving PN weaning.

## 2 Methods

This paper is a narrative review for which we systematically and extensively searched PubMed, Embase, and Web of Science databases. The search included all studies published up to April 2026, using the following keywords, alone or in combination: “teduglutide or Gattex or Revestive” and “children or pediatric” and “short bowel syndrome”. The searches were filtered to include only studies published in English. This study was conducted under the Preferred Reporting Items for Systematic Reviews and Meta-Analysis (PRISMA) statement. This selection process ensures that this review is built on a foundation of relevant research. Most of the data are sourced from clinical trials and real-world studies, which provide a high level of evidence and support relatively robust conclusions. We thoroughly examined each included article, assessing the methodologies, results, and implications. This process enabled us to summarize current knowledge in the field of achieving PN weaning, identify existing research gaps, and lay a foundation for future clinical applications.

The final studies that were included in the discussion of this narrative review include randomized controlled trials, non-randomized studies and cross-sectional studies. The quality of randomized controlled trials were assessed using the Cochrane risk of bias tool (22). We assessed the methodological quality of non-randomized studies for the risk of bias using the Methodological Index for Non-randomized Studies(MINORS) (23). The methodological quality of cross-sectional studies were evaluated using the Joanna Briggs Institute (JBI) critical appraisal tool (24). Two investigators independently conducted quality assessments. Disagreements were resolved by consensus or, if necessary, by involving a third evaluator.

## 3 Results

### 3.1 Study selection and research flow chart

558 records were retrieved from PubMed, Embase, and Web of Science databases. After removing duplicates, 435 potentially eligible articles were identified. Ultimately, 12 research were included in our study (PRISMA in the Supplementary Files). Representative studies on achieving intestinal autonomy using TED in children with SBS are summarized in Table 1 and the general demographic characteristics of the clinical studies in Table 2.

**Table 1:** Clinical studies on the impact of TED treatment duration and age on achieving intestinal autonomy outcomes in children.

References	Type of study	Number of participants use TED	Duration of TED	The age at TED therapy initiation	Number of participants achieve PN weaning
Carter et al. (25)	phase III trial	37 children	12 weeks	Median aged 3.0 (1,14)years	At week 12, 4 patients achieved PN independence: 3 in the 0.05 mg/kg/day cohort (weeks 4, 8, and 12) and 1 in the 0.025 mg/kg/day cohort (week 11).
					At week 16 (4 weeks post-discontinuation), 2 of these 4 patients resumed PN, while 2 sustained PN independence.
Kocoshis et al. (26)	phase III trial	50 children	24 weeks	0.025 mg/kg/day [*n* = 24, mean age 7 (4)years],	At week 24：Five achieve PN weaning.
				0.05 mg/kg/day [*n* = 26, mean age 6 (4)years]),	Two in the 0.025 mg/kg/day group (weeks 8 and 10)；
				9 received SOC [mean age 6 (5)years	Three in the 0.05 mg/kg/day group (weeks 14, 21, and 21)
Muñiz et al. (27)	Non-interventional multicentre cohort study	24 children	24 weeks	Mean age of 9.7 ± 4.6 years	6 (25%; 95%CI: 9.7–46.7) in the pediatric cohort were weaned from PS support at the 24-week assessment
	(retrospective and prospective study)				
Lambe et al. (15)	Single-center clinical trial	25 children	a median of 38 weeks of therapy	Mean age 9.4 (7.3–12)years	Eight（32%） achieve PN weaning within 48 weeks of therapy
Laura et al. (16)	Multicenter non-interventional retrospective cohort study	7 children	at least 6 months，median of 1.07 years	Mean age 7 (1.26–13.39) years	At 12-month follow-up, 14.29% of children (*n* = 1/7) had weaned off HPN;
					By the end of the study, complete PN discontinuation was achieved in 50.0% of children (3/6)
Boluda et al. (28)	Prospective study	17 children	All patients completed 12 months of therapy but 1	Age 12–121 months	A total of 12/17 patients achieved parenteral independence：3 patients after 3 months, 4 at 6 months, and 5 after 12 months of treatment
Chiba et al. (20)	Two open-label phase 3 studies and 1 extension study	7 infants and 8 children in core studies，2 infants and 7 children in the extension	Mean teduglutide exposure of infants was 305.3 ± 186.3 days (in core study) and 133.0 ± 50.9 days (inextension study);	Infants aged 4 to 12 months and children aged 1 to 15 years	Two children (25.0%) attained enteral autonomy(at week 12 and 28)，both sustaining independence through week 48. No infants achieved enteral autonomy
			Mean exposure of children was 164.4 ± 11.1 days (in 24-week core study) and 169.3 ± 0.8 days (in 48-week extension study).		
Guz-Mark et al. (29)	Multicenter retrospective cohort study	13 children	Between 3 and 51months [median (IQR) of 18 (12–30) months], with 77% (*n* = 10/13) of patients treated for longer than 1 year	Mean age 6 (4.7–7)years	Two responders (15.4%) achieved enteral autonomy after 12 and 27 months of therapy
Martínez et al. (17)	Multicenter retrospective cohort study	33 children	1.5 years (IQR 0.52–3.2; range 0.25–6.7)	7.5 years (IQR 4.8–12.4; range 2.5–18)	Complete PN discontinuation without detriment to nutritional status was achieved in 4 out of 33 patients at month 3, in 6 out of 29 patients at month 6, and in 8 out of 19 patients at month 12 of TED treatment. Among responders, 12 patients(36%) successfully discontinued PN.
Germán-Díaz et al. (18)	Prospective observational study	31 children	The median duration of treatment was 19 months (IQR 12–36), with 23 patients (74%) receiving treatment for over 1 year and 9 patients (29%) for more than 3 years	2.3 years (IQR 1.4–4.9)	Among the responders, 9 patients (29%) achieved enteral autonomy, four patients required more than 12 months of treatment to achieve adaptation, and three patients required over 20 months of treatment
Wales et al. (21)	Long-term extension studies (retrospective and prospective study)	78 children	96 weeks	Mean age 4 (0.5–15)years	In the any-TED group, 19.2% and 21.8% of patients achieved enteral autonomy at 48 and 96 weeks, respectively
Josey et al. (19)	Single-center retrospective analysis	27 children	mean 120.8 weeks	Mean age 8.9 years	8 patients (30%) reached enteral autonomy at mean 71.57 (SD = 56.37) weeks

TED, teduglutide; PN, parenteral nutrition; SOC, standard-of-care.

**Table 2:** The general demographic characteristics of the clinical studies on the impact of TED treatment duration and age on achieving intestinal autonomy outcomes in children.

References	The geographic distribution of the studies	Number of participants use TED in Race	The cause of SBS in the clinical studies	Number of participants at the age at TED therapy initiation	Number of participants use TED in sex	Number of participants colon-in-continuity
Carter et al. (25)	At 17 sites in the United States and the United Kingdom		Necrotizing enterocolitis:6			33
		White:30	Midgut volvulus:13	1–3y:17	Male:25	
		Black:4	Intestinal atresia:7	4–12y:17	Female:12	
		Asian:1	Gastroschisis:12	13–17y:3		
		Other:2	Other:3			
Kocoshis et al. (26)	At 24 centers in North America and Europe		Necrotizing enterocolitis:8			44
		White:37	Midgut volvulus:16	1–11y:46		
		Black:6	Intestinal atresia:3	12–16y:4	Male:35	
		Asian:2	Gastroschisis:20	17–18y:0	Female:15	
		Other:5	Hirschsprung disease:2			
			Other:1			
Muñiz et al. (27)	All the nutritional support SBS specialists prescribing teduglutide in Argentina were invited to participate. The patients were assessed for inclusion and enrolled in this real-world study by 14 specialized physicians in 10 sites	/	Volvulus:8			22
			Intestinal atresia:7			
			Gastroschisis:5		Male:5	
			Injury/traumatic:1	3–17y:24	Female:19	
			Necrotizing enterocolitis:1			
			Other:2			
Lambe et al. (15)	One intestinal failure rehabilitation center in France	/	Long segment Hirschsprung disease：6			/
			Gastroschisis:5	5–8y:12		
			Atresia:4	9–11y:7	Male:16	
			Midgut volvulus:7	12–17y:6	Female:9	
			Necrotizing enterocolitis:3			
Laura et al. (16)	The data were collected from the electronic from the chronological medical records of patients followed long-term by their specialists (gastroenterology/hepatology and pediatrics facilities in the Slovak Republic)	/	Volvulus:3			/
			Necrotizing enterocolitis:1			
			Intestinal atresia:1	mean age 7 (1.26–13.39) years	Male:5	
			Gastroschisis, intestinal atresia:1		Female:2	
			Gastroschisis:1			
Boluda et al. (28)	From 8 Spanish centers	/	Necrotizing enterocolitis:6			/
			Volvulus:3			
			pediatric intestinal pseudo-obstruction:1			
			Gastroschisis:2	age 12–121 months	17 children	
			Hirschsprung disease:2			
			Intestinal atresia:3			
Chiba et al. (20)	At 7 study sites across Finland, France, and the United Kingdom and 6 sites in Japan		Infants：			
			Necrotizing enterocolitis:1		Infants：	
		Infants：	Midgut volvulus:1		Male:5	
		White:4	Intestinal atresia:1	4–5m:2	Female:2	
		Asian:3	Gastroschisis:3	6–12m:5	Children:	Infants:7
		Children：	Other:1	1–18y:8	Male:7	Children:8
		Asian:8	Children:		Female:1	
			Midgut volvulus:6			
			Intestinal atresia:1			
			Gastroschisis:1			
Guz-Mark et al. (29)	In 8 medical centers across Israel	/	Volvulus:2			/
			Necrotizing enterocolitis:5			
			Atresia:1			
			Vascular accident:1	mean age 6 (4.7–7)years	Male:7	
			Gastroschisis:2		Female:6	
			Diaphragmatic hernia:1			
			Hirschsprung disease:1			
Martínez et al. (17)	In specialized centers in Argentina	/	Gastroschisis:8			/
			Atresia:10			
			Necrotizing enterocolitis:1	7.5 years (IQR 4.8–12.4; range 2.5–18)	Male:21	
			Neonatal Volvulus:6		Female:12	
			Non-neonatal Volvulus:3			
			Others:5			
Germán-Díaz et al. (18)	With the participation of seven hospitals from all over the country in Spain	/	Necrotizing enterocolitis:11			/
			Midgut volvulus:6			
			Gastroschisis:6	2.3 years (IQR 1.4–4.9)	Male:20	
			Long segment Hirschsprung disease:4		Female:11	
			Atresia:2			
Wales et al. (21)	The pooled data from two phase 3 long term extension clinical trials(NCT02949362, NCT02954458) were analyzed up to 96 weeks in United States and United Kingdom	White:59	Gastroschisis: 26	mean age 4 (0.5–15)years		69
		Black or African American:9	Midgut volvulus: 24			
		Asian:3	Necrotizing enterocolitis: 12		Male:54	
		Other:2	Intestinal atresia: 5		Female:24	
		Not allowed based on local regulations:5	Hirschsprung's disease: 1			
			Volvulus and ischemic bowel:1			
Josey et al. (19)	At a single center in United States	/	Gastroschisis: 10	mean age 8.9 years		23
			Necrotizing enterocolitis: 7		Male:17	
			Hirschsprung's disease: 3		Female:10	
			Volvulus:3			
			Other:4			

TED, teduglutide; Y, year; SBS, Short Bowel Syndrome.

### 3.2 Risk of bias assessment

In terms of bias risk in included studies, different research use different evaluation tools. The quality of included randomized controlled trials (20, 21, 26, 28) were assessed by the Cochrane risk of bias tool ((A) Summary of Bias Risk for Each Trial. (B) Risk of Bias Graph for All Included Trials in the Supplementary Files). In terms of blinding of outcome assessment, two studies (20, 21) were assessed as high risk. Among nonrandomized studies, 3 (15, 25, 28) were of moderate quality(MINORS score 13/16,17/24,13/16). One or two questions JBI Cross-sectional Tools were unclear in all cross-sectional studies of the review (16–19, 27, 29). For instance, all cross-sectional studies in strategies to deal with confounding factors stated were unclear. The four studies (16–18, 27) of the review were considered the criteria for inclusion unclear. Furthermore, most of the original clinical studies focused on adults, and there is insufficient data on children, which limits the generalizability of the conclusions.

### 3.3 Pediatric clinical trials

#### 3.3.1 The duration of TED is among 12 to 24 weeks

The initial 12-week, open-label, multicenter, phase 3 study by Carter et al. (25), was conducted across 17 sites in the United States and the United Kingdom (25). It enrolled patients with SBS-IF of a median aged of 3.0(1,14) years, who received different doses of teduglutide or standard-of-care (SOC). All patients required parenteral nutrition (PN) and showed minimal or no progress in advancing enteral nutrition (EN). A total of 54 patients were screened, of whom 42 were enrolled and sequentially assigned to one of four cohorts: three teduglutide dose groups (0.0125 mg/kg/day [*n* = 8], 0.025 mg/kg/day [*n* = 14], or 0.05 mg/kg/day [*n* = 15]) or a standard-of-care (SOC) group (*n* = 5). At week 12, four patients receiving teduglutide achieved PN independence: three in the 0.05 mg/kg/day cohort (at weeks 4, 8, and 12) and one in the 0.025 mg/kg/day cohort (at week 11). At week 16 (4 weeks post-discontinuation), two of these four patients resumed PN, while the other two sustained PN independence. Although the study period was relatively short, teduglutide treatment was associated with a trend toward PN independence in children with SBS-IF, especially at the higher doses of 0.025 and 0.05 mg/kg/day. Whether sustained PN independence can be achieved appears to depend on the duration of early teduglutide therapy. Additional follow-up research will be required to generate more conclusive data.

Kocoshis et al. conducted a 24-week, phase III trial in children with SBS-IF who received teduglutide or SOC across 24 centers in North America and Europe (26). Of 59 enrolled patients, 50 elected teduglutide(0.025 mg/kg/day [*n* = 24, mean age 7(4)years] or 0.05 mg/kg/day [*n* = 26，mean age 6(4)years]), while 9 received SOC [mean age 6(5)years]. All patients completed the 24-week treatment and subsequent 4-week follow-up. Five treated patients(10%) achieved enteral autonomy by week 24:two in the 0.025 mg/kg/day group (at weeks 8 and 10), and three in the 0.05 mg/kg/day group (at weeks 14, 21, and 21). Notably, three of these five patients achieved autonomy after 12 weeks of treatment. No patients in the SOC group attained enteral autonomy. Further investigation is warranted to evaluate whether extended teduglutide treatment sustains reductions in parenteral support or maintains efficacy after treatment discontinuation.

In 2025, a non-interventional multicentre cohort study in Argentina included 21 adult and 24 children with SBS(mean age 9.7 ± 4.6 years) who were dependent on parenteral support (PS) and received teduglutide at 0.05 mg/kg/day (30). Overall, 6 children(25%; 95%CI: 9.7–46.7) achieved PS weaning at the 24-week assessment.

These studies suggest that the result of achieving PN weaning may be influenced by both the initiation time and the duration of TED.

#### 3.3.2 The duration of TED is among 24 weeks to 1 year

In 2023, Lambe et al. presented an open-label, single-center clinical trial involving children[*n* = 25, mean age 9.4(7.3–12)years] with SBS-IF treated with 0.05 mg/kg/day teduglutide (a median of 38 weeks of therapy) (15). The treatment facilitated enteral autonomy within 48 weeks in 32% of children. Comparative analysis of children who achieved complete PN weaning (*n* = 8) before the end of the study vs. those remaining PN-dependent revealed significant baseline differences in PN dependence:the weaned cohort had lower PN volume, lower PNDI, and fewer infusions per week. The baseline PN dependence and oral intake emerged as significant predictors of treatment success, which may help identify patients more likely to benefit from teduglutide therapy. More importantly, the children with SBS enrolled in this study were older, had spent more time on parenteral nutrition(2 years) and had shorter remnant bowel (small bowel length <80 cm). The preservation of the colon is essential for energy circulation and GLP-2 analogues may further enhance overall intestinal absorption (31, 32).

A 2024 multicenter real-world, non-interventional retrospective cohort study evaluated teduglutide treatment for adult and children with SBS (16). Data were collected from all children and adult patients diagnosed with SBS who received teduglutide treatment at least 6 months(the median duration of teduglutide treatment for children was 1.07 years). The patients were treated with the recommended dose of 0.05 mg/kg/day of teduglutide. This study included 16 patients treated with teduglutide: 7 children[mean age 7(1.26–13.39)years] and 9 adults. The primary outcome was graded home parenteral nutrition(HPN) weaning status (defined as discontinuation of all parenteral nutrition and hydration support), which was assessed at 12 weeks, 6 months, and 12 months. At the 12-month follow-up, 14.29% of children (*n* = 1/7) had weaned off HPN. By the end of the study, complete HPN discontinuation was achieved in 50.0% of children (3/6).

Seventeen children(aged 12–121months) with SBS-IF received subcutaneous teduglutide (0.05 mg/kg/day) across several Spanish hospitals (28). This was the first real-life experience published for children. All but 1 patient completed 12 months of therapy. Twelve of the 17 patients achieved parenteral independence: 3 patients after 3 months of treatment, 4 at 6 months, and 5 after 12 months. These findings demonstrate that teduglutide is effective in promoting parenteral independence in children with SBS, with a significant proportion achieving enteral autonomy over time. The gradual increase in weaning rates highlights the importance of sustained treatment.

Current findings reveal a delayed, incremental response pattern across pediatric studies, emphasizing that therapeutic effects of TED manifest gradually rather than acutely. These studies also underscores the importance of long-term follow-up to capture the full therapeutic benefits of teduglutide.

#### 3.3.3 The duration of TED is more than 1 year

The 2023 analysis, which included 3 trials, provides the first enteral autonomy data on teduglutide for SBS-IF infants under 1 year old (20). Two open-label phase 3 trials (NCT03571516, NCT02980666) and a 24-week extension study (NCT03268811) evaluated the safety and efficacy of teduglutide (0.05 mg/kg/day) in infants and children with SBS-IF. NCT03571516 is a 24-week study that randomized infants to receive either teduglutide or SOC; NCT02980666 is a 24-week study that enrolled infants and children to exclusively receive teduglutide; NCT03268811 is a 24-week extension study that extended treatment for participants who completed NCT02980666, for a total treatment duration of up to 48 weeks. These studies included infants aged 4 to 12 months and children aged 1 to 15 years.12 infants(*n* = 5, SOC; *n* = 7, TED) and 8 children(*n* = 8, TED)were enrolled in the core studies, and 2 infants(*n* = 2, TED) and 7 children(*n* = 7, TED) were enrolled in the extension study. Mean teduglutide exposure was 305.3 ± 186.3 days for infants in the core study, and 133.0 ± 50.9 days for infants in extension study. Mean exposure was 164.4 ± 11.1 days for children in the core study, and 169.3 ± 0.8 days for children in the extension study. The results showed that no infants achieved enteral autonomy. In children, two children (25.0%) attained enteral autonomy(one at week 12 and another at week 28), and both sustained this independence through week 48. The child who achieved autonomy at week 12 discontinued teduglutide during the extension study, suggesting sustained therapeutic effects after treatment. The second child's delayed response (at week 28) suggests that longer teduglutide exposure may be necessary for some patients to achieve enteral autonomy.

A multicenter retrospective cohort study evaluated 13 children [comprising all eligible children, mean age 6 (4.7–7))years] with SBS-IF treated with teduglutide across eight Israeli medical centers (29). The treatment duration of teduglutide ranged from 3 to 51months [median (IQR) 18 (12–30) months], and 77% (*n* = 10/13) of patients received treatment for longer than 1 year. Two patients (15.4%) achieved enteral autonomy after 12 and 27 months of therapy, respectively.

A multicenter retrospective cohort study evaluated 33 children with IF/SBS treated with TED across nine specialized Argentine centers between January 2017 and August 2023 (17). All patients received subcutaneous TED at 0.05 mg/kg/day. Median age at starting TED was 7.5 years (IQR 4.8–12.4; range 2.5–18). The median treatment duration was 1.5 years (IQR 0.52–3.2; range 0.25–6.7). Specifically, 15 patients were treated with TED for ≤12 months, 6 patients received treatment between 12 and 24 months, and 12 patients were treated for over 24 months. Overall, 12 out of 33 patients (36%) achieved response to TED throughout the follow-up period. Complete PN discontinuation without detriment to nutritional status was achieved in 4 out of 33 patients at month 3, in 6 out of 29 patients at month 6, and in 8 out of 19 patients at month 12 of TED treatment. Among responders, median time from the initiation of TED treatment to PN independence was 8.16 months (IQR 2.8–20.2; range 3–41) in the 12 patients who successfully discontinued PN.

A prospective observational study was conducted by the Intestinal Failure Working Group of the Spanish Society of Pediatric Gastroenterology, Hepatology and Nutrition (SEGHNP) from August 2017 to January 2023 (18). This study enrolled children with intestinal failure who had received PN ≥90 days and received subcutaneous teduglutide (0.05 mg/kg/day) for ≥3 months. The study cohort consisted of 31 patients from seven hospitals. The median age at treatment initiation was 2.3 years (IQR 1.4–4.9). The median duration of treatment was 19 months (IQR 12–36), with 23 patients (74%) receiving treatment for over 1 year and 9 patients (29%) for more than 3 years. Overall, 9 patients (29%) achieved enteral autonomy, with a median treatment duration of 6 months (IQR 4.5–22).

In a pooled *post hoc* analysis in 2024, the long-term (96-week) safety, efficacy, and predictive factors of teduglutide response were evaluated in children with SBS-IF (21). Data were derived from two open-label extension studies (NCT02949362, NCT02954458). The analysis included 85 patients:the TED-exposed group[*n* = 78, mean age 4(0.5–15)years] received teduglutide in parent and/or extension studies, while the control group[*n* = 7, mean age 4(0.7–17)years] never received teduglutide (NTT/NTT). In the any-TED group, 19.2% and 21.8% of patients achieved enteral autonomy at 48 and 96 weeks, respectively. In contrast, in the NTT/NTT group, 14.3% of patients achieved enteral autonomy at both 48 and 96 weeks.

In 2025, the study performed a single-center, retrospective analysis of teduglutide use(0.05 mg/kg/d) for a mean of 120.8 weeks in 27 children(mean age 8.9 years) with SBS (19). As a result, 8 patients (30%) successfully weaned off intravenous fluids at a mean of 45.35 (SD = 59.28) weeks, and reached enteral autonomy at a mean of 71.57 (SD = 56.37) weeks.

These results emphasize the significance of treatment duration. Due to the varying response times of patients to the treatment, some patients who should have experienced delayed reactions might be prematurely terminated from the treatment, and the extended treatment time may be associated with enhanced effectiveness.

## 4 Discussion

### 4.1 The result of achieving PN weaning

A 2022 systematic review of 14 studies conducted in patients under 18 years of age with SBS-IF revealed that among 223 patients treated with teduglutide, a total of 36 (16%) achieved enteral autonomy after a median of 24 weeks of treatment (IQR: 24–48 weeks) (14). 152 and 38 patients were treated with 0.05 and 0.025 mg/Kg/d of teduglutide, 8 received either 0.125 or 0.20 mg/Kg/d, the dose was not speciﬁed for 17 patients, and 25 were in the SOC arm. The included studies had heterogenous results. This proportion is much lower than most reports published after 2022 (15–19, 27) and similar to the results of two open-label extension studies (21). For example, in the 2023 single-center study,32% (8/25) of patients achieved enteral autonomy after a median of 38 weeks of therapy (15). In a 2024 Argentine multicenter retrospective cohort study,36% (12/33) of patients achieved PN discontinuation during the follow-up period(4 out of 33 patients achieved PN discontinuation at month 3, 6 out of 29 patients at month 6, and 8 out of 19 at month 12 of TED treatment) (17). In a 2024 Spanish prospective observational study,29% (9/31) of patients achieved enteral autonomy, with a median treatment duration of 6 months (IQR 4.5–22)(four patients required more than 12 months of treatment, and three patients required over 20 months of treatment) (19). In a 2025 two-center retrospective cohort study, 36% (5/14) of patients achieved weaning from PN (2 discontinued PN within 6 months of treatment initiation, and 1 of these 2 attained enteral autonomy (18). The remaining 3 children weaned from PN between 6 and 12 months of therapy, and 2 of these 3 achieved enteral autonomy). These results indicate that new research has made progress.

### 4.2 The associations between the baseline characteristics of patients and the achievement of enteral autonomy

The Guz-Mark study in 2022 found no significant associations between baseline parenteral support (PS) requirement of patients and the achievement of enteral autonomy including PN volume and energy at baseline (*P* = 0.7 and *P* = 0.3, respectively) and enteral intake at baseline (*P* = 0.3) (29). However, according to the Lambe study, significant baseline differences in PN dependence (lower PN volume, lower PNDI and fewer infusions per week) are associated with the probability of intestinal adaptation (15). This finding is similar to the Muñiz study, which reported that baseline PS requirement was inversely associated with weaning from PS(*P* = 0.025) (27). Recently, a multicenter cohort study(2025) collected retrospective and prospective data of 104 children with SBS undergoing TED treatment in 7 Europe countries (including Italy, Spain, Croatia, Germany, France, Israel, Portugal) (33). The median age of the cohort was 6.7 years old (IQR: 3.6–10.4).21 children achieved PN weaning after 12 months (cumulative incidence: 22%, 95% CI 15%–31%). The study found that pretreatment PN calories <35 kcal/kg/day (*p* = 0.044) predicted complete PN weaning. The Josey study proposed that baseline PNDI can be used to predict the achievement of enteral autonomy (19). No successful PS weaning was observed among patients with baseline PNDI values exceeding 53%; for the 8 patients with baseline PNDI values ranging from 15% to 53%, no successful weaning was observed either, indicating that a higher PNDI may predict unsuccessful weaning. Furthermore, subgroup analysis comparing patients below and above 5 years of age at baseline indicated that older patients experienced more significant decreases in PS dependency (including PS volume, PS, PNDI, and nonprotein calories provided by PS) compared to patients under 5 years of age. These results may guide the indication for the use of teduglutide in children with SBS.

In addition to the aforementioned studies on PS requirement, a 2025 multicenter cohort study, found that pretreatment citrulline ≥14 μmol/L (*p* = 0.047) predicted complete PN weaning, as well as early and steady increases in haemoglobin and a citrulline rise in the first 6 months of treatment (*p* = 0.014 and *p* = 0.044 respectively) (33). This may reflect enhanced absorption capacity of the residual bowel for iron and/or vitamin B12, or excessively high blood concentration after active weaning. This finding is highly consistent with real-world results of French adults (34), monitoring changes in hemoglobin and citrulline from baseline to 6 months of treatment may help determine whether TED treatment should be continued. The Josey study proposed that discontinuing lipid supplementation during teduglutide treatment can help predict the achievement of enteral autonomy (19). During treatment, the dosage of lipid used was significantly reduced. All patients who continued to require PS were unable to discontinue lipid use, which indicates that discontinuing lipid use may mean that the patients are about to achieve enteral autonomy.

However, no statistically significant differences existed in age, remnant small bowel length, SBS anatomical classification, intestinal absorption rate, citrulline concentrations, or endogenous GLP-2 concentrations in the Lambe study (15). No associations were demonstrated in the study (29) between response to teduglutide and patients' baseline characteristics, including gender (*P* = 0.4), history of prematurity (*P* = 0.7), residual small bowel length (*P* = 0.6), the presence of an ileocecal valve (*P* = 0.9), and age at treatment initiation (*P* = 0.8). The Muñiz study also analyzed the selected variables (study cohort, anatomy type, small bowel length, and time from PS to start of treatment) and found that they were not associated with weaning from PS (27). Importantly, the majority of patients in this study had a colon in continuity, unlike most patient cohorts in the United States and Europe. However, this study did not find any correlation between the presence of colon in continuity and PS autonomy, as observed in other studies (15). The study on adult with SBS pointed out that the presence of a stoma and the absence of an ileocecal valve were positive predictors of the early response to teduglutide (35).

These findings suggest that children with SBS who have significant baseline differences in PN dependence (lower PN volume, lower PNDI and fewer infusions per week) can benefit the most from teduglutide treatment. Furthermore, lipid discontinuation, haemoglobin levels, and citrulline levels may also predict complete PN weaning.

### 4.3 The initiation time of TED

The optimal initiation time of teduglutide administration is of crucial importance for the successful treatment of children with SBS–IF. Current evidence indicates that the outcomes of early vs. delayed starting times are different. The rate of parenteral support weaning in children who received teduglutide(25.0%) at the 24-week assessment in the 2025 non-interventional multicentre cohort study by Muñiz et al. (27) was higher than the rate(10%) reported in the 24-week clinical trial by Kocoshis et al. (26). However, in the 2025 cohort, the mean age at treatment initiation was relatively late(9.7 vs. 6 years). This mean age was similar to that reported by Lambe et al. (15), which was 9.4 years. Lambe et al. considered that children treated early require continuous use of teduglutide after PN weaning, and suggested that to account for the impact of physiological intestinal adaptation after extensive resection (36), a minimum age and time on PN required is 3 years. They emphasize the importance of distinguishing between physiological adaptation and pharmacologically induced effects. Furthermore, treating children in the early stages of life may require continuous teduglutide therapy even after PN weaning. These findings imply that delayed initiation may align with the natural physiological intestinal adaptation following extensive bowel resection, potentially reducing the need for prolonged teduglutide therapy after PS weaning.

Conversely, emerging data support the feasibility and potential benefits of earlier intervention. A prospective observational study(2025) reported that ten children with SBS(aged between 1.4 and 11.6 years) in the Czech Republic received a daily dose of 0.05 mg/kg/day teduglutide (37). Three patients (30%) achieved enteral autonomy after four, six and twenty months of treatment, respectively. Their ages at treatment initiation were 1.5, 5.7 and 11.6 years. Some recent studies also recommend early initiation of treatment at a median age of 2.3–4.0 years (18, 21, 25). It is necessary to balance the potential benefits of early teduglutide intervention against the uncertainties associated with long-term use of teduglutide in young children.

Notably, preliminary evidence has extended the therapeutic window to infants and younger children (aged <12 months or weighing <10 kg). Two open-label phase 3 trials conducted in 2023 (20)(NCT03571516, NCT02980666) and a 24-week extension study (NCT03268811) included infants aged 4 to 12 months.12 infants(*n* = 5, SOC; *n* = 7, TED)were included in core studies, and 2 infants(*n* = 2, TED)were included in the extension study. The results showed no infants achieved enteral autonomy. However, by breaking through the limitation of existing studies that mostly focus on adults and older children, this research provides the first evidence of TED efficacy in the infant group. The 2025 phase 3, open-label study enrolled Japanese children with SBS-IF weighing less than 10 kg, who received 0.05 mg/kg/day teduglutide subcutaneously (38). Three patients -an 11-month-old infant, a 1-year-old child and a 2-year-old child completed the study, with a mean teduglutide exposure duration of 48.9weeks. At the end of the treatment(the overall observation duration was 60.9 weeks), compared with the baseline, both the mean PS volume (13.1%) and the mean PS calorie intake (46.6%) showed clinically meaningful reductions. None of the patients were weaned off PS during the study. Since available data on teduglutide use in infants aged below 12 months, especially those weighing less than 10 kg, these findings offer valuable insights for SBS treatment. Nevertheless, these observations must be interpreted cautiously, as clinical data in infants remain extremely limited, and the long-term safety profile of chronic teduglutide use in young children remains incompletely defined.

In conclusion, teduglutide is a powerful tool for promoting enteral autonomy in children with SBS-IF, but its optimal initiation timing needs to be determined based on the individual characteristics of each patient, such as baseline conditions. Instead of following a uniform recommendation, an individualized treatment timing strategy should be adopted. Delayed initiation (9–10 years) aligns with natural physiology and has demonstrated high PS weaning rates. Early initiation (1.5–4.0 years) is feasible and may offer benefits in selected patients. Treatment in infants under 12 months remains experimental and should be confined to specialized research settings. The central clinical challenge is to respect spontaneous intestinal adaptation while not missing the window for pharmacological support.

### 4.4 The duration of TED

Treatment duration is a critical determinant of therapeutic success with teduglutide in children with SBS-IF, and cumulative evidence demonstrates that prolonged therapy is associated with enhanced intestinal adaptation, higher rates of PS weaning, and greater clinical benefit. In the Lambe trial, PN weaning occurred after a median of 38 weeks of therapy, with progressive PN volume reduction observed in most patients after weeks 24 and 36 (15). The observed delay in PN weaning suggests that the therapeutic effects of teduglutide may manifest gradually over time, highlighting the importance of sustained treatment. In the Laura trial, duration of TED was at least 6 months (median of 1.07 years) (16). At 12-month follow-up, 14.29% of children had weaned off HPN;by the end of the study, complete HPN discontinuation was achieved in 50.0% of children. In the Boluda trial, up to 70% of patients who continued 1 year of teduglutide treatment were able to wean off PN (28). In summary, among children, the delayed weaning effect suggests that sustained treatment is important for optimal outcomes.

However, we observed a heterogeneous response trajectory: the Guz-Mark study reported a wide range of time to response, Two responders (15.4%) achieved enteral autonomy after 12 and 27 months of therapy respectively, which is somewhat inconsistent with the previously reported positive findings (29). These results imply that the absence of a primary response after 1 year may indicate treatment failure. Nevertheless, for patients who achieve an initial response after 1 year, long-term treatment with teduglutide may deliver substantial improvements when therapy is prolonged.

The Marta trial(29%) reported very similar results to the Lambe trial (15), which achieved enteral autonomy within 48 weeks in 32% of children (18). What differs is the response timeline in the Marta trial:among the 9 patients (29%) who achieved enteral autonomy, four patients required more than 12 months of treatment to achieve adaptation, and three patients required over 20 months of treatment. These results reinforce the importance of treatment duration that extended treatment durations may be associated with enhanced effectiveness. This study is the first to systematically report the continuous therapeutic effect of teduglutide in children with SBS for up to 3 years, providing the currently longest available data on the effectiveness of teduglutide use in children. It is particularly worth noting that 29% of the patients maintained their therapeutic effect after more than three years of treatment, which has significant reference value for establishing long-term medication norms for children.

Consistent predictors of favorable outcomes reinforce the importance of treatment duration. In an Argentine multicenter retrospective cohort study, longer TED duration was the sole statistically significant predictor of PN independence (17). Notably, the PN-dependent cohort may include latent responders requiring extended therapy. In the 2025 open-label study, compared with the baseline, the decline in PS volume and PS caloric intake at the end of the second cycle was greater than that at the end of the first cycle (38). This might indicate a delayed response to teduglutide, which is consistent with previously published studies (20). These studies have shown that the longer the duration of teduglutide treatment, the greater the clinical benefits. It has also been confirmed that patients with SBS-IF who have colon-in-continuity are more likely to benefit from a longer treatment duration. Most patients experienced a significant reduction in PS after one year of treatment (35).

Long-term data extending beyond 2 years remain limited but informative. Few previous studies have reported the efficacy of TED beyond 2 years (21, 28). The retrospective study describes over 3 years responses among patients with childhood-onset SBS, comparing adult(*n* = 4, median age of 29 years) and children (*n* = 14, median age of 8 years) (39). PN requirements in adults decreased by up to 54.6%, with one patient achieving PN independence. Children showed gradual reductions of up to 31.9%, with none achieving PN independence. The lower rates of PN withdrawal likely result from more severe anatomical deficits, such as ultra-short bowel length. In 2025, Japan reported the first known case documenting TED use for >6 years in a child with SBS (40). The patient was enrolled in a TED clinical trial at the age of 5 years and continued treatment. During >6 years of TED therapy, the patient achieved complete weaning from PN. Although the response was slower than in previously reported cases, this case highlights the potential of TED to support long-term intestinal adaptation and emphasizes the importance of long-term treatment strategies for managing severe SBS in children.

Given the variability in patient response times to treatment, some patients who might have achieved a late response could have their treatment prematurely discontinued. A 2026 systematic review included 23 studies reporting on glucagon-like peptide-2 analogue use in adults with intestinal failure (41). This review showed teduglutide demonstrated a positive incremental effect on the proportion of patients with SBS-IF achieving enteral autonomy, with response rates increasing from 13% (95% CI: 6%–25%) at 6 months to 31% (95% CI: 23%–40%) after ≥2 years of treatment. Additional long-term research is also required to confirm the incidence of late response. The 2024 pooled *post hoc* analysis therefore recommends continuing treatment for a minimum of one year before assessing the drug's effectiveness (21).

As the duration of TED treatment increases, the high cost is another factor that needs to be considered when using TED over parenteral nutrition. In a recent study in Paris on costs of SBS-IF treatment, the study compare a group of children with SBS-IF on home PN treated with subcutaneous TED with another group of children who were not treated TED. A gradual reduction in the budget of PN and its complications that were significantly lower in the group of patients treated with TED. Nevertheless, the model was not cost-effective when the price of TED was included in the expenses (42). Therefore, it is important to discuss the use of strategies to reduce costs.

Furthermore, security is also something that deserves particular attention. In the teduglutide-treated groups, most patients experienced adverse event were mild or moderate. The most common TEAEs in the teduglutide-treated groups were pyrexia, vomiting, abdominal pain and abdominal distension. A very few TEAEs were moderate or severe(abdominal pain) (20, 21).

In summary, the therapeutic benefits of teduglutide in children with SBS-IF are time-dependent and cumulative. While some children achieve enteral autonomy within 6–12 months, a substantial proportion require 12–36 months or longer of continuous treatment to attain maximal PN weaning. Prolonged treatment duration is the strongest predictor of enteral autonomy, and premature discontinuation at 6–12 months may incorrectly label late responders as treatment failures. Therefore, it is recommended to continue the treatment for at least one year before re-evaluating the drug's effectiveness. Heterogeneous response timelines mandate individualized, long-term management rather than fixed short-term trials. While long-term data remain limited, available evidence supports extended therapy in slow responders.

## 5 Conclusion

SBS is a complex disease that is the main cause of intestinal failure in children, and it presents a significant therapeutic challenge for this population. Teduglutide is the first GLP-2 analog developed and marketed for SBS-IF, and it is currently FDA approved for the management of PN, or intravenous fluid, dependent children with SBS who are over one year of age. This literature review examines several recent studies on the use of teduglutide for children with SBS. The collated data collection may help identify which patients are most likely to benefit from treatment, an important consideration given the high cost of teduglutide.

According to the most up-to-date studies, TED can help up to more than 30% of patients achieve complete enteral autonomy, a rate much higher than the 16% reported in the systematic review of 14 studies involving children conducted in 2022. The optimal timing to initiate treatment for children with SBS remains under debate, and variations in study designs underscore the need for individualized timing strategies rather than a universal recommendation. Teduglutide requires long-term administration in the treatment of SBS-IF to achieve the best therapeutic effect. Studies have shown that longer treatment durations are associated with more significant clinical benefits. Due to differences in individual patient response times, some late responders may lose out on the therapeutic benifits from prematurely discontinuing the medication. Therefore, it is recommended to continue treatment for at least one year before re-evaluating the drug's effectiveness.
