# Supplementary material for: Optimizing teduglutide treatment regimens in children with short bowel syndrome
Source: Front Pediatr. 2026 Jul 29;14:1898958. doi: 10.3389/fped.2026.1898958 (PMC13461579; doi:10.3389/fped.2026.1898958)
Supplement: Supplementary file 1 [file Supplementaryfile1.docx]

Supplementary files

Records removed before screening:

Duplicate records removed (n=123)

Records identified from:

Databases (n=558)

**Identification**

Records excluded(n=390)：

Review articles (n=266)

Case report (n=31)

Irrelevant studies (n=94)

Records screened

(n=435)

**Screening**

Reports assessed for eligibility

(n=44)

Reports excluded(n=32)：

Abstract only (n=18)

Clinical Trial (n=10)

Other language (n=1)

Multicenter results (n=1)

Outcome indicators were not used (n=2)

Studies included in review

(n=12)

**Included**

FIGURE 1.PRISMA diagram for the process of the search and study selection
